# Supplementary material for: Feeding pattern and associated factors among children aged 6–23 months in the Tahtay Maichew district, northern Ethiopia
Source: PLOS Glob Public Health. 2026 Feb 6;6(2):e0003949. doi: 10.1371/journal.pgph.0003949 (PMC12880657; doi:10.1371/journal.pgph.0003949)
Supplement: Attachment — Submitted filename: Response to Reviewers.docx [file pgph.0003949.s003.docx]

**Point-by-Point Response to Reviewers**

**Reviewer 1**

**Introduction**

Dear Reviewer,

We thank you for your review and valuable feedback on our manuscript. We have made revisions according to your requests, as outlined below.

**Reviewer comment 1:**
The study is significant, but the authors should pay attention to the concerns.
**Abstract**
Specify types of multivariable analysis.

**Response:** We thank you for your comment. We have specified it as “a multivariable binary logistics regression analysis **(See page 2, line 26 in the revised manuscript).**

**Reviewer comment 2:** How was the endpoint variable assessed?

**Response:** The endpoint variable (minimum meal frequency of children aged 6-23 months) was assessed by the 24-hour recall method asking the mothers (caretakers) how many times the child consumed solid, semisolid, or soft foods in the day preceding the survey **( see page 2, line number 24).** We also have elaborated this in the **operational definitions** subsection **(see page 11, line numbers 156-161).** Thank you for the comment.

**Reviewer comment 3:**
**Results**
Specify the wealth index.

**Response:** Thank you. We have specified it based on your comment as the “rich wealth index” **(see page 2, line number 29)**.

**Reviewer comment 4:**
**Conclusion**
Expunge the word "recommendation"

**Response:** Thank you, we have removed it now.

**Reviewer comment 5:** Lines 31-33 Statements were similar to the ones in the results.

**Response:** We have revised it now: "The proportion of children who did not receive the minimum meal frequency was significantly high. Poor wealth index, lack of growth monitoring and promotion follow-up, and low maternal knowledge were associated with inadequate meal frequency. In conclusion, our findings underscore the need for targeted interventions to enhance maternal knowledge, growth monitoring and promotion services, and socioeconomic support. By addressing these factors, we can potentially improve meal frequency among children, thereby contributing to their health and development” **(see page 3 lines 33-39).**

**Reviewer comment 6:**

**Main body
Methods**
The methodology has major defects.
Source population, study population, inclusion, and exclusion criteria were missed.

**Response:** Thank you for your valuable comment. We have amended the methodology. We also have stated the source population, study population, inclusion criteria, and exclusion criteria under the subsections of **population** and **eligibility criteria (see pages 6-7, line numbers 89-99).**

**Reviewer comment 7:** What is the justification for the 4% margin error? DE?

**Response:** Generally, we use a lower margin of error to improve precision especially when the prevalence of the outcome is lower (45% in our case). So, we decided to use a 4% margin of error to improve the precision of the estimates and also increase the sample size. We used a design effect because our sampling procedure had more than one stage.

**Reviewer comment 8:** How many HH assets were assessed?
How did you include HH assets for further analysis (PCA)?

**Response:** Thank you for your valuable comment. To clarify, "The wealth index was assessed by collecting data on 24 household assets, including livestock, household equipment, annual cereal production, vehicle ownership, agricultural land ownership, and housing conditions (roofing, walls, and flooring). Each of these assets was recorded as either 0 (not owned) or 1 (owned). We then generated factor scores using principal component analysis. Finally, we summed these factor scores and categorized them into three groups: poor, medium, and rich” **(see page 11, lines 162-167).**

**Reviewer comment 9:** Mention the response variable before data processing and analysis.

**Response:** Thank you, we have mentioned the response variable in the **measurements** and **operational definitions** sub-sections before **data processing and analysis (see page 10 line number 144, and page 11 line numbers 156-161)**.

**Reviewer comment 10:** What was the maximum value of VIF?

**Response:** The maximum value of VIF was 1.

**Reviewer comment 11**: What was the justification for a p-value < 0.1 for candidate predictors in the final model?

**Response:** We chose a p-value threshold of <0.1 for our bivariate logistic regression to ensure the robustness of results, taking into account our sample size and the number of variables we have. **Using a higher threshold, like p-value <0.25, could lead to false positives (Type I errors).** This means, we might find statistically significant associations between variables when there is no true association. This situation may arise when we include too many variables in our model relative to our sample size. By opting for a p-value threshold of <0.10**, we aimed to reduce the risk of overfitting.**

**Reviewer comment 12:** Why did you use mean and SD for age?

**Response:** Because age is a continuous variable, and the data were normally distributed.

**Reviewer comment 13:**

Ethical issues
Confidentiality issues were not mentioned

**Response:** it is stated now in the **Ethics Approval and Consent to Participate** section **(see page 13, lines 195-201).**

**Reviewer comment 14:**

Results
Do not interpret the findings

**Response:** Thank you, we have made corrections according to your comment.

**Reviewer comment 15:**

Discussion
The discussion is very clumsy.
English writing and editorial issues are not optimal.

**Response:** We have revised the discussion and made corrections to the English writing and editorial problems. Thank you for this comment.

**Reviewer 2**

**Introduction**

Dear Reviewer,

We thank you for your review and valuable feedback on our manuscript. We have made revisions according to your comments, as outlined below.

**Reviewer comment 1:**

Meal frequency and its associated factors among children aged 6-23 months in the Tahtay Maichew district, northern Ethiopia

First of all, I appreciate the work you did but I have the following main questions

Why you are targeting infants or children aged 6-23 months?

**Response:** Thank you for this valuable comment. We targeted children aged 6-23 months, because children in this age group are within the critical 1000 days, a period of window opportunity for children’s growth and health. This is the time of rapid body and brain development. Therefore knowing the feeding situation specifically the meal frequency of children in this age range and the factors affecting it can be helpful for our informed interventions.

**Reviewer comment 2:** What is the novelty of your study since many studies done with similar topics in the study area and country?

**Response:** Thank you for this comment. There are indeed studies conducted in Ethiopia, but most of them were done in regions out of Tigray, particularly in the southern and northwestern parts of the country. In addition, large-scale surveys like the Ethiopian Demographic and Health Survey, while valuable, may overlook local socioeconomic, and cultural variations. Therefore, our study aimed to provide more specific and localized evidence on child meal frequency and its associated factors in the Tahtay Maichew district, thereby informing targeted interventions.

**Reviewer comment 3:** In general, the methods part needs major revision
**Abstract**: The background information ¨ The term “meal frequency” describes the adequacy of complementary feedings among children¨ it would be better if you focused on or included the severity or magnitudes of the problems or the Gap.

**Response:** We thank you for this comment. We have made modifications in the revised manuscript accordingly **(see page 1, line numbers 14-21).**

**Reviewer comment 4:** Results: is not written clearly, so would you re-write again

**Response:** Thank you for your comment. We have written it again with modifications to increase its clarity **(see pages 14-18)**.

**Reviewer comment 5:** Methods: We conducted a community-based cross-sectional study among 981 mothers with index children aged 6-23 months. It would be better if changed to A community-based cross-sectional study was conducted among 981 mothers with index children aged 6-23 months.

**Response:** We have changed it based on your comment **(see page 2 line numbers 22-23)**. Thank you.

**Reviewer comment 6:** Conclusion and recommendation: would you write it based on your findings

**Response:** We have amended it based on our findings as follows **“**The proportion of children who did not receive the minimum meal frequency was significantly high. Poor wealth index, lack of growth monitoring and promotion follow-up, and low maternal knowledge were associated with inadequate meal frequency. In conclusion, our findings underscore the necessity for targeted interventions aimed at enhancing maternal knowledge, growth monitoring and promotion services, and socioeconomic support. By addressing these factors, we can potentially improve meal frequency among children, thereby contributing to their health and development” **(see page 3 lines 33-39).**

**Reviewer comment 7:** Introduction: Para 2 and 3 are redundant as they have no direct relevance to the current objectives of the study, and are not required for the audience of journals dealing with PLOS Global Public Health. The introduction section is too short and not clear, and it still is unable to appropriately justify neither the need for this study nor the target age group.

**Response:** Thank you for your comment. We have revised the whole introduction section according to your comments. We have tried to make it clearer, and we also addressed the significance of the study concerning the title, the age group, and the study area **(see pages 4-5)**.

**Reviewer comment 8:** Methods: The recruitment period for this study was from 22/1/2018 to 30/2/2018. How is it possible to collect such much data within specified data, n=981? I don’t think so? Would you check it again?

**Response:** It was from 22/1/2018 to 30/3/2018, we made a typing error. It is corrected now **(see page 6, line number 83)**. Thank you for the comment.

**Reviewer comment 9:** Sample size determination: Why 4% margin of error, and a nonresponse rate 10%? Why not 5% and NRR=5%?

**Response:** Generally, we use a lower margin of error to improve precision especially when the prevalence of the outcome is lower (45% in our case). So, we decided to use a 4% margin of error to improve the precision of the estimates and also increase the sample size. Regarding the nonresponse rate, in the context of the study area child feeding is culturally sensitive and mothers don’t usually feel comfortable talking about it, that’s why we anticipated a 10% nonresponse rate.

**Reviewer comment 10:** Initially, out of the seventeen kebeles in the Tahtay Maichew district, eight kebeles were chosen using the lottery method. How did you select eight of them?

**Response:** We selected those eight kebeles using simple random sampling.

**Reviewer comment 11:** **Did you any sampling techniques or randomization? if how?**

**Response**: Thank you for this comment. We have stated our sampling technique and randomization as follows “Initially, out of the 17 kebeles in the Tahtay Maichew district, 8 were chosen using a simple random sampling method. Subsequently, the total sample size was distributed proportionately among the selected kebeles based on the number of children aged 6-23 months found from community-based demographic and health-related information registration prepared by health extension workers. Following this, a rapid census was conducted on the selected kebeles 07 days before the actual data collection to identify the target households. Finally, child-mother pairs were selected from each kebele using a systematic random sampling technique after giving a code for each household that has a child aged 6-23 months” **(See pages 7-8, line numbers 109-119)** and **(Figure 1, on page 9, line numbers 120-134)**.

**Reviewer comment 12:** What is your sampling frame?

**Response:** A rapid census was conducted on the selected kebeles 07 days before the actual data collection to identify households with children aged 6-23 months and that was our sampling frame.

**Reviewer comment 13:** What does mean Kebelle?

**Response:** Kebele is the smallest administrative unit in Ethiopia, we have mentioned it in brackets in the study design and setting section **(see page 6, line number 87-88)**.

**Reviewer comment 14:** Did you use PPS? how?

**Response:** Yes, the total sample size was distributed proportionately among the selected kebeles based on the number of children aged 6-23 months found from community-based demographic and health-related information registration prepared by health extension workers. Thank you for your comment.

**Reviewer comment 15:** Using a systematic random sampling technique versus a sampling frame? So how did you see those two things?

**Response:** Thank you for your crucial comment. It would have been ideal if we used a simple random sampling, but the challenging geographic terrain and widely dispersed households in the study area made it difficult to use it given the limited time and resources we had.

**Reviewer comment 16:** Your sampling procedure is not clear, please write it clearly.

**Response:** We have written it again in a clearer way according to your suggestion **(see pages 7-8, line numbers 109-119)** and **(Figure 1, on page 9, line numbers 120-134)**. Thank you.

**Reviewer comment 17:** Would you include operational definitions like?
¬ PCA?
¬ Knowledge good or poor?
¬ Maternal Social capital good or poor?
¬ Received minimum meal frequency?
¬ Etc

**Response:** We thank you for your comment. We have included a sub-section for operational definitions and operationally defined the above variables now **(see pages 11-12, line numbers 155-177).**

**Reviewer comment 18:** What was your outcome variables?

**Response:** The outcome variable was the minimum meal frequency of children aged 6-23 months.

**Reviewer comment 19:** In data management and analysis, did you check the normality distribution of your data? How?

**Response:** Yes, we have checked the normality of our data for the continuous variables using the skewness test and P-P plot **(see page 12, line numbers 182-183)**. We thank you for this comment.

**Reviewer comment 20:** Write what you did for categorical and continuous variables.
For example
The mean age [±SD] of the children was 13.7 ±4.59 months.
The mean age [±SD] of the mothers was 29.74 ±6.66 years.
The mean family size [±SD] was 4.75 ±1.7 persons.
Not nothing mentioned in the methods part

**Response:** Thank you, we have included it according to your comment as follows **“**During descriptive analysis, continuous variables such as the age of children, age of mothers, and family size were summarized using means and standard deviations after checking the normality of data for the continuous variables using the skewness test and P-P plot. Frequencies and percentages were calculated for categorical variables.” In the **Data Management and Analysis** sub-section **(see page 12, line numbers 181-184)**.

**Reviewer comment 21:** Results: Table 1: Socio-demographic and socio-economic characteristics of participants
¬ Do cross tab for Tables 1 and 2

**Response:** We have done cross-tabulation for both tables **(see pages 14-15 and page 16 for both tables respectively)**. Thank you for your valuable suggestion.

**Reviewer comment 22:**

¬ Mothers age in years change it to age of mother (yrs)
¬ Age of index child in months change it to Age of child (mo)
¬ Family size in persons change it to family size
¬ Etc , do the other like this

**Response:** We thank you for the comment. We have modified it accordingly in the tables.

**Reviewer comment 23:** Do you think the Husband's educational status is important?

**Response:** Yes, the husbands’ educational status may be associated with the households’ economic capacity, and their knowledge about child feeding, which could in turn influence the feeding of their children. Thank you.

**Reviewer comment 24:** Did you assess GMP?

**Response:** Yes, we have assessed GMP.

**Reviewer comment 25:** In the bivariate analysis: According to the bivariable logistic regression analysis, eight variables, namely, wealth index, ANC follow-up, place of delivery, growth monitoring follow-up, health extension worker home visit, mother’s knowledge of child feeding, media exposure, and mother’s social capital in the last 12 months, were significantly associated with meal frequency at 95% CL (p ≤ 0.1). Why the reason you only select those variables p≤0.1, and why not p≤0.25?

**Response:** We chose a p-value threshold of <0.1 for our bivariate logistic regression to ensure the robustness of results, taking into account our sample size and the number of variables we have. **Using a higher threshold, like p-value <0.25, could lead to false positives (Type I errors).** This means we might find statistically significant associations between variables when there is no true association. This situation may arise when we include too many variables in our model relative to our sample size. By opting for a p-value threshold of <0.10**, we aimed to reduce the risk of overfitting.**

**Reviewer comment 26:**

Discussion
¬ The discussion needs to be focused towards the main objectives. The functional significance of the present results may be elaborated.
¬ Please include the strengths and limitations of the study
¬ what is the clinical implication of your findings?

**Response:** We thank you for these comments. We have revised the whole section of the discussion, addressing all these vital comments **(see pages 19-20)**.

**Reviewer comment 27:** Conclusion: Would you rewrite it based on your findings?

**Response:** We have revised it now: "The proportion of children who did not receive the minimum meal frequency was significantly high. Poor wealth index, lack of growth monitoring and promotion follow-up, and low maternal knowledge were associated with inadequate meal frequency. In conclusion, our findings underscore the need for targeted interventions to enhance maternal knowledge, growth monitoring and promotion services, and socioeconomic support. By addressing these factors, we can potentially improve meal frequency among children, thereby contributing to their health and development” **(see page 3 lines 33-39).**

**Reviewer comment 28:** References: References need to be written in the style recommended by the journal PLOS Global Public Health.
For example, if you take reference numbers 1 and 2 are written wrongly
1. Organization, W.H., Infant and young child feeding: model chapter for textbooks for medical
students and allied health professionals. 2009: World Health Organization. Available from: include it
2. Organization, W.H., Indicators for assessing infant and young child feeding practices: part 1:
definitions: conclusions of a consensus meeting held 6-8 November 2007 in Washington DC, USA. 2008: World Health Organization . Available from:include it

**Response:** Thank you for raising this issue. We have modified the reference section following your suggestion and the journal’s recommendation.
