# Supplementary material for: Feeding pattern and associated factors among children aged 6–23 months in the Tahtay Maichew district, northern Ethiopia
Source: PLOS Glob Public Health. 2026 Feb 6;6(2):e0003949. doi: 10.1371/journal.pgph.0003949 (PMC12880657; doi:10.1371/journal.pgph.0003949)
Supplement: Attachment — Submitted filename: Response_to_Reviewers_auresp_2.docx [file pgph.0003949.s004.docx]

**Point-by-Point Response to Reviewers**

**Reviewer 1**

**Introduction**

Dear Reviewer,

We appreciate your review and helpful feedback on our manuscript. We have made the requested revisions, as outlined below.

**Reviewer comment 1:** Meal frequency and its associated factors, among children aged 6-23 months in the Tahtay Miachew District, northern Ethiopia.

The authors have attempted to respond to the concerns; however, it is not fully addressed as requested.

**Response:** We thank you again for your constructive guidance. We have made further amendments to address the remaining concerns and strengthen our manuscript.

**Reviewer comment 2:** Abstract
Line 14: Replace introduction by background.

**Response:** Thank you, we have corrected it accordingly.

**Reviewer comment 3:** Line 19: Indicate the research gap rather than stating ‘little is known about meal frequency...

**Response:** Thank you very much for this comment. We have avoided the vague term “little is known” and stated the research gap clearly **(See page 2, lines 17-21 in the revised manuscript).**

**Reviewer comment 4:** Specify outcome assessment in the methods

**Response:** We have specified the way the outcome variable was assessed **(See page 2, lines 24-25).**

**Reviewer comment 5:**

Introduction
Line 71: indicate the research gap clearly.

**Response:** Thank you for this comment. We have indicated the research gap with more clarity now **(See page 5, lines 70-78).**

**Reviewer comment 6:**

Methods
There are still methodological flaws

**Response:** Thank you for this valuable feedback. We have revised our methodology to address the identified flaws according to your comments. The details are outlined below.

**Reviewer comment 7:** Lines 90-99: Restate it in paragraph form.

**Response:** Thank you for this comment. We have written it in paragraph form **(See page 6, lines 90-95).**

**Reviewer comment 8:** The authors did not specify the threshold for how HH assets were selected for further analysis.

**Response:** We thank you a lot for giving us this comment. We have specified the thresholds as follows “In the analysis, we assessed the suitability of the variables for Principal Component Analysis (PCA) using the Kaiser-Meyer-Olkin (KMO) and communality values. The KMO threshold value was 0.8 indicating sampling adequacy, while the communality values were 0.6 suggesting the variable is well represented by the extracted components. After that, variables with eigenvalues >1 were selected for further analysis as this indicates the corresponding principal component explains more variance.” **(See page 10, lines 161-166)**

**Reviewer comment 9:** The authors are recommended to redo analysis by taking a threshold, a p value < 0.05, as the authors did not present scientifically convincing argumentative evidence.

**Response:** Thank you for your comment. We have re-analyzed the data using a p-value threshold of <0.05 for both bivariate and multivariable logistic regression analysis. We used a backward elimination analysis method, giving us the adjusted odds ratio and confidence interval of only the significant variables. While the significant variables remain unchanged, there are changes in the adjusted ratios and confidence intervals and we have updated the table and the narration accordingly **(See page 16, lines 231-238 and page 17, Table 3).**

**Reviewer comment 10:** The limitations of the report should be fully explained, and overstated conclusions are expected to be revised.

**Response:** We thank you for this comment. We have addressed this concern now. We have expanded the limitations to make it more comprehensive, adding limitations like geographically limited scope of the study, and day-to-day meal frequency variations which can’t be captured by single 24-hour recall. Additionally, we have revised the overstated conclusions to reflect the scope and limitations of our study **(See page 19, lines 278-283 and page 20, lines 285-290)**.
