# Supplementary material for: Feeding pattern and associated factors among children aged 6–23 months in the Tahtay Maichew district, northern Ethiopia
Source: PLOS Glob Public Health. 2026 Feb 6;6(2):e0003949. doi: 10.1371/journal.pgph.0003949 (PMC12880657; doi:10.1371/journal.pgph.0003949)
Supplement: Attachment — Submitted filename: Response_to_Reviewers_auresp_3.docx [file pgph.0003949.s005.docx]

**Point-by-Point Response to Reviewers**

**Introduction**

Dear Editor,

Thank you so much for your review and helpful feedback on our manuscript. We have revised the manuscript following your suggestions, as outlined below.

**Comment 1:** The study has been done meticulously in field but needs major revision in the write up of manuscript

**Response 1:** Thank you very much for your valuable comment. We have carefully reviewed the write-up and made corrections based on your comments and suggestions.

**Comment 2:** Title: Meal frequency or dietary assessment?

**Response 2:** Thank you for the crucial suggestion. You are right that the title needs improvement. We have changed it to “Feeding pattern and associated factors among children aged 6-23 months in the Tahtay Maichew district, northern Ethiopia.” We replaced meal frequency with feeding pattern for it is the closest MESH term and is catchier.

**Comment 3:** Study participants: Why mothers and not parent?

**Response 3:** Thank you for your comment. At this young age (6-23 months), it is mothers who stay close to the children, breastfeeding, providing complementary food, and generally caring for them. That is why we chose mothers as the study participants.

**Comment 4:** Elaborate on poverty level, any conflicts in study area

**Response 4:** Thank you for this important suggestion. We have written about the poverty level in the region in the study design and setting subsection as: “The Tigray region has a higher poverty level because its economy mainly depends on agriculture, is drought-prone, receives limited rainfall, and farmers have small landholdings that limit their production.” **(See page 6, lines 86-88)** Regarding conflicts, the region was peaceful when this study was conducted. There was a war, but it broke out in late 2020 and ended in 2022.

**Comment 5:** Key words: Select up to 10 key words from MESH

**Response 5:** We thank you for this valuable suggestion. We have selected six key words which are related to our title from MESH. **(See page 3, line 38)**

**Comment 6:** Line no 54-56: If things go wrong during this time we only have little opportunity to correct them later and the consequences may be devastating concerning the health and future of the child - Rewrite in professional English

**Response 6:** Thank you so much for bringing this to our attention. We have now corrected it to: “If nutritional deficiencies occur during this time, they can have serious consequences for the child’s health and development, with limited chances for correction later” **(See page 4, lines 44-46)**

**Comment 7:** Introduction: Mention about exclusive breastfeeding and continued breastfeeding rates in the study area

**Response 7:** Thank you for highlighting this issue. We have now included the two key points as follows: “Infant and Young Child Feeding practices such as exclusive breastfeeding and continued breastfeeding until the age of two years are at rates of 58% and 76% in Ethiopia, respectively.” **(See page 4, lines 56-57)**

**Comment 8:** Line no 83: Duration of study mention only year and not date

**Response 8:** Thank very much for this comment. We have removed the dates and corrected it as: “The study was conducted in 2018” **(See page 6, lines 82-83)**

**Comment 9:** The weather conditions of the district are semi-highland: Check the necessity of this statement

**Response 9:** Thank you for raising this point for us. The sentence was unnecessary, and we have removed it.

**Comment 10:** Line no 89: Populations or study participants?

**Response 10:** Thank you for your comment. We have changed it to study participants now. **(See page 6, line 89)**

**Comment 11:** Add ethical approval statement in methods in the beginning itself

**Response 11:** Thank you for your suggestion. We have moved the Ethical approval statement from the end of the methods section to the beginning as per your suggestion. **(See page 5, line 73)**

**Comment 12:** International Code of Marketing of Breast-milk Substitutes: Adherence to the code in the study area needs to be mentioned in methods or introduction

**Response 12:** Thank you very much for this valuable comment. We have addressed the two important issues you raised in the introduction section, as follows: “Ethiopia is committed to the International Code of Marketing of Breast-milk Substitutes and has had a legal framework supporting the code’s principles since 2016. However, there are limitations on the regulation of follow-up formula and growing-up milk. On the other hand, breastfeeding is culturally encouraged in Ethiopia, and most mothers, especially in rural areas, are willing to breastfeed, even though there are concerns regarding its appropriateness.” **(Page 4-5, lines 58-62)**

**Comment 13:** Elaborate operational definitions and variables description is not required - Please rewrite concisely

**Response 13:** Thank you very much for bringing this up. We have now shortened the variable description and written the operational definitions concisely, merging them in the measurement subsection, following your suggestion**. (See page 9, line 123)**

**Comment 14:** The results need more elaboration on Breastfeeding, types of foods consumed- with breakup of macro and micronutrients, consumption of junk food, screen time, anthropometric status of children, any illness

**Response 14:** Thank you for these important comments. We have clarified in the result section about exclusive and current breastfeeding as: “In addition, 70.5% of the children were exclusively breastfed for the first six months, while 94.5% of them were currently breastfed.” **(See page 11, lines 167-168)** We also added a paragraph about the types of foods children consumed, stating: “The majority of the children (96.94%) were provided with grains, tubers, and roots, while only a small percentage received flesh foods, fruits, and vegetables.” Additionally, we included a figure showing food groups rich in macro- and micronutrients, along with the percentage of children who consumed them. Foods high in micronutrients are represented by orange bars, while the others are represented by light blue. **(See page 13, lines 171-174)** However, we didn’t collect data on junk food consumption or screen time because, in the rural settlements of the study area, people do not have access to junk foods, and mobile phones are limited. Furthermore, mobile phones are culturally prohibited for children under 2 years or even up to around 15 years old. We were unable to collect anthropometric data due to logistical and financial constraints, and we also did not gather information on illnesses.

**Comment 15:** Presentation of results in very basic, with less efforts from authors to regroup variables and make the tables in presentable fashion

**Response 15:** Thank you very much for providing these important comments. The presentation of the result and tables indeed needed improvement. We merged the variable and category columns in Tables 1 and 2 into a single column. We adjusted the borders and added spacing between numbers and percentages to improve their appearance. Additionally, we regrouped variables from different subsections of the results. For example, the subsection titled “Knowledge, media exposure, and social capital related characteristics of participants” has been removed and combined with the "Socio-demographic characteristics" subsection. We also re-categorized the birth interval into two categories, as <36 and ≥ 36 months. **(See Tables 1, 2, and 3, on pages 12, 14, and 16)**

**Comment 16**: Tables and presentation of data can be improved by making it concise

**Response 16:** Thank you for your comment on this issue. We have made revisions to improve the results and table presentation, making it more concise. We also eliminated redundancy by ensuring that data presented in the narration is not repeated in the tables. We have presented the age of mothers, the age of children, and family size as the mean ± SD, to make the table more succinct. Table 3 is made more concise by removing the columns that show frequencies and percentages, which are already presented in tables 1 and 2. **(See Tables 1, 2, and 3, on pages 12, 14, and 16)**

**Comment 17:** Wherever you are comparing meal frequency add a p value and statistic. Mention details in legend

**Response 17:** Thank you for bringing this to our attention. We have added p-values to Tables 1 and 2. Additionally, we included p-values for the crude and adjusted odds ratios, along with their respective 95% confidence intervals in Table 3, as detailed in the legend. P-values and statistics are also mentioned in the results and abstract narratives. **(See Tables 1, 2, and 3, on pages 12, 14, and 16) and (Page 15, lines 189-196)**

**Comment 18:** What is the MAM and SAM treatment protocol in the study area - are there any food security, feeding programs in the study area

**Response 18:** Thank you for these valuable comments. We have elaborated on the management of MAM and SAM, food security, and feeding issues in the discussion section as follows: “Regarding treatment, uncomplicated severe acute malnutrition (SAM) is treated in the outpatient therapeutic program (OTP). Meanwhile, complicated SAM is managed at the stabilization center (SC) as an inpatient treatment. Conversely, moderate acute malnutrition (MAM) is treated through the targeted supplementary feeding program (TSFP). Additionally, food security and feeding programs include the Productive Safety Net Program (PSNP), emergency food assistance for vulnerable households, and the General Food Distribution (GFD).” **(See page 19, lines 258-264)**

**Comment 19:** What does the health system in study area do to ensure regular growth monitoring? What are the measures to ensure universal health coverage of malnutrition prevention in the study area?

**Response 19:** Thank you so much for raising these issues. We have addressed the issue of growth monitoring in the discussion as: “Moreover, regular Growth Monitoring and Promotion (GMP) is carried out in the study area by health extension workers who perform GMP for children under two years old every month and use the family health card to track all children. It is also conducted at health centers integrated with the Integrated Management of Newborn and Childhood Illness (IMNCI), despite limitations caused by a lack of equipment, inaccurate measurements, and low service utilization.” **(See pages 18-19, lines 242-247)** We have also elaborated on the coverage of malnutrition prevention in the response to comment 20 below.

**Comment 20:** What are the existing health system interventions to prevent malnutrition in the study area?

**Response 20:** We thank you for this comment. We have written on the interventions to prevent malnutrition as: “In summary, in the study area, efforts are being made to prevent malnutrition through nutrition education, micronutrient supplementation, and deworming. The National Nutrition Program II (NNP II) and the Ethiopian food and nutrition policy are also in place to implement nutrition-sensitive and nutrition-specific interventions through a coordinated multi-sectoral approach.” **(See page 19, lines 254-257)**

**Comment 21:** Discussion is not written with the public health angle of the problem studied - it needs to be rewritten using the suggestions given by me

**Response 21:** We appreciate your valuable comment. We have rewritten the whole discussion, emphasizing the public health perspective of the problem according to your suggestion. For instance, we have elaborated on the public health implications of not receiving adequate meal frequency as: “While this is a significant portion, it also indicates that nearly one-third (32%) of children did not receive the appropriate meal frequency. This suggests that suboptimal feeding during this critical period of rapid growth and development increases the risk of macronutrient undernutrition, micronutrient deficiencies, and impaired cognitive and physical development, potentially leading to long-term health and economic burdens for individuals and society.” **(See page 17, lines 201-206)** We have added more on this in the response to comment 22 below.

**Comment 22:** You can elaborate on best practices from other resource limited settings in which dietary intake was ensured among under 5 children

**Response 22:** We are grateful for this valuable comment. As you suggested, we have elaborated on best practices from other similarly resource limited areas and have stated it as: **“**Moreover, it emphasizes the importance of strengthening social protection programs such as the productive safety net program, promoting income-generating activities for poorer families, and generally focusing on poverty alleviation in the area, as these interventions are effective in other studies from low-income settings” **(See page 18, lines 229-232)** Additionally, we have added other practices as: “This suggests a need for public health interventions, such as nutrition education and behavioral change communication, to enhance their understanding of age-appropriate meal frequency and feeding practices in general. Such efforts can be delivered through various contact points, including antenatal and postnatal care, delivery, and community health days.” **(See page 19, lines 249-253)**
