# Supplementary material for: Feeding pattern and associated factors among children aged 6–23 months in the Tahtay Maichew district, northern Ethiopia
Source: PLOS Glob Public Health. 2026 Feb 6;6(2):e0003949. doi: 10.1371/journal.pgph.0003949 (PMC12880657; doi:10.1371/journal.pgph.0003949)
Supplement: Attachment — Submitted filename: Response_to_Reviewers_auresp_4.docx [file pgph.0003949.s006.docx]

**Point-by-Point Response to Reviewers**

**Introduction**

Dear Editor,

We thank you so much for your effort and time to review and provide helpful feedback for the improvement of our manuscript. We have revised the manuscript once again following your suggestions, as outlined below.

**Comment 1:** While there was response to the reviewer's comments, Authors have attempted only superficially attempted to address the reviewers' concerns. Substantial revision of the paper has not been undertaken.

**Response 1:** We sincerely appreciate your constructive feedback. We have revised the manuscript again and made further improvements so that it reflects your valuable input.

**Comment 2:** There was Northern Ethiopian Conflict which could or not have impacted the food security.

**Response 2:** Thank you for raising this important concern that will help to improve the clarity of the study. As we stated in our response to the earlier revision, the Northern Ethiopian conflict broke out in 2020 after this study was already conducted.

**Comment 3:** Public health aspects such as health system, female literacy, age of marriage, spacing, family size etc needs to be focussed.

**Response 3:** We discussed the health system in relation to the GMP service provided in the study area as follows: “Regular growth monitoring and promotion (GMP) is carried out in the study area by health extension workers who perform GMP for children under two years old every month and use the family health card to track all children. It is also conducted at health centers incorporated with the integrated management of newborn and childhood illness (IMNCI), despite limitations caused by a lack of equipment, inaccurate measurements, and low service utilization.” **(See page 17, lines 215-220).** We also focused on female literacy in the discussion, as “Mothers’ good knowledge of child feeding was also a key factor influencing whether their children received adequate meal frequency. This suggests a need for public health interventions, such as nutrition education and behavioral change communication, along with improving women's literacy to enhance their understanding of feeding practices.” **(See page 17, lines 222-225)** Additionally, we highlighted birth spacing and family size when discussing the association between birth preparedness and meal frequency, as stated: “Having a birth preparedness plan was also associated with adequate meal frequency, which may be because individuals who plan for their birth are also more likely to have appropriate birth spacing and a smaller family size.” **(See page 17, lines 227-229)** We didn’t collect data on the age of marriage.

**Comment 4:** MDD-IYCF as per UNICEF could have been measured but it is not focssed in the paper.

**Response 4:** We appreciate your valuable comment. We addressed this issue in the discussion section as follows: “On the other hand, among the children who had adequate meal frequency, only 43.4% achieved an adequate level of dietary diversity. This finding highlights an important gap; even when children are fed adequately during the day, the foods they eat may still lack variety. In other words, meeting meal frequency does not necessarily ensure a nutritionally balanced diet among children. This underscores that our interventions should not only promote feeding frequency but also emphasize diverse feeding.” **(See page 16, lines 193-198)**

**Comment 5:** Kindly rewrite the manuscript, strengthen introduction, results and discussion and submit.

**Response 5:** Thank you so much for your suggestion. We have rewritten the manuscript and strengthened the introduction, results, and discussion sections in response to the comments and suggestions you provided, both in this revision and in the previous one.

**Comment 6:** Key words need to be upto 10

**Response 6:** We have added some more keywords; we have ten of them now. We thank you for your suggestion.
