# Supplementary material for: Feeding pattern and associated factors among children aged 6–23 months in the Tahtay Maichew district, northern Ethiopia
Source: PLOS Glob Public Health. 2026 Feb 6;6(2):e0003949. doi: 10.1371/journal.pgph.0003949 (PMC12880657; doi:10.1371/journal.pgph.0003949)
Supplement: Attachment — Submitted filename: Response_to_Reviewers_auresp_5.docx [file pgph.0003949.s007.docx]

**Point-by-Point Response to Reviewers**

**Introduction**

Dear Editor,

Thank you very much for your effort and time spent reviewing and providing helpful feedback to improve our manuscript. We have revised the manuscript once again in response to your suggestions, as outlined below.

**Comment 1:** Abstract: Grammatical errors need to be corrected. Mention study site

**Response 1:** Thank you for your comment. We have corrected the grammatical errors in the abstract as you suggested. We also included the study site. **(See)**

**Comment 2:** Key words: Add MESH terms such as Malnutrition, stunting etc

**Response 2:** We appreciate this comment. We have now included MESH terms such as malnutrition, stunting, and wasting, as you recommended.

**Comment 3:** Study setting section needs to be added in methods

**Response 3:** Thank you for this suggestion. The study setting was previously included in the 'Study design, setting, and population' subsection. We have now moved it to its own section, as you recommended.

**Comment 4:** Discussion can be strengthened by adding details about food security of the region

**Response 4:** We appreciate this valuable comment. We have enhanced the discussion by adding information about the region's food security in the paragraph that covers the association between wealth index and meal frequency, as follows: “In Tigray, most households do not meet the daily recommended calorie intake. Tigray experiences drought, characterized by limited, unreliable, and variable rainfall, resulting in repeated crop failures and asset depletion. The shortage of grazing land and water reduces livestock productivity, which ultimately affects per-capita food consumption and food security.” **(See)**

**Comment 5:** Conflict of interest and author contributions section need to be mentioned

**Response 5:** Thank you for bringing this issue to our attention. We have added the conflict of interest and author contributions sections. **(See)**
